# 5-Aminolevulinic Acid Phosphate as an Immune System Enhancer Along with Vaccination Against SARS-CoV-2 Virus Infection: An Open-Label, Randomized Pilot Study

**DOI:** 10.3390/life15060953

**Published:** 2025-06-13

**Authors:** Norbert Berenzen, Riyadh Rehani, Andrea Ebeling, Marcus Stocker, Motowo Nakajima

**Affiliations:** 1 Photonamic GmbH & Co. KG, Eggerstedter Weg 12, 25421 Pinneberg, Germany; riyadhrehani@hotmail.com (R.R.); a.ebeling@photonamic.de (A.E.); m.stocker@photonamic.de (M.S.); 2 SBI Pharmaceuticals Co. Ltd., Izumi Garden Tower, 1-6-1 Roppongi, Minato-ku, Tokyo 106-6013, Japan; motnakaj@sbigroup.co.jp

**Keywords:** 5-aminolevulinic acid, COVID-19, IgG, immune booster, 5-ALA, vaccine, SARS-CoV-2

## Abstract

Previous studies have shown that 5-aminolevulinic acid phosphate together with sodium ferrous citrate, which is marketed as a food supplement, appears to be an important metabolic regulator in depleted T cell metabolism. Therefore, it was hypothesized that its administration in subjects vaccinated against COVID-19 could enhance their immune system. Therefore, the aim of our proof-of-concept study was to determine the safety (by adverse events monitoring) and the tolerability (by subject questionnaires) and to investigate immune-boosting properties (by Immunoglobulins) in which 200 subjects were randomized in a ratio of 1:1 within 2 arms. In the intervention arm, the study product was administered together with the vaccines Covishield or Covaxin, and up to 21 days thereafter with a 150 mg daily dose, whereas in the control arm, the subjects were vaccinated only. No safety issues were detected, and the evaluation of the subject questionnaires showed no limitation of the well-being, which confirms the excellent tolerability of 5-aminolevulinic acid phosphate with sodium ferrous citrate. Moreover, the ‘Change in Immunoglobulin G levels’, although statistically insignificant, showed strong signals of its immune supportive potential. However, further studies are recommended to verify the results.

## 1. Introduction

Since the coronavirus outbreak in December 2019, various drugs and vaccines against coronavirus disease 2019 (COVID-19) have received marketing authorization, mostly with emergency approval [1]. With these drugs, symptoms can usually be effectively treated, and in vaccinated individuals, the course of infection is predominantly mild. In addition, herd immunization led to the pandemic no longer being considered a global emergency as of 5 May 2023. Now, COVID-19 has been globally acknowledged as a persistent disease. Therefore, the pandemic would no longer meet the definition of a “Public Health Emergency of International Concern.” However, the World Health Organization (WHO) stresses that it still exists [2]. Many individuals become infected or reinfected with SARS-CoV-2 in spite of being vaccinated [3]. COVID-19 has had a disproportionate impact on the elderly and those with comorbidities, with higher rates of severe illness and hospitalization.

Studies have demonstrated that Immunoglobulin G (IgG) levels can drop quickly in subjects vaccinated with various vaccines currently on the market. This was, e.g., evident in the case of mRNA vaccines [4], which are comparable to viral vector vaccines such as Covishield, with which most of the subjects in the present study were vaccinated. The decrease in IgG antibodies could be associated with decreasing immunogenicity. A strong correlation between Severe Acute Respiratory Syndrome Coronavirus 2 (SARS-CoV-2) spike-specific CD4+ T cells and IgG antibodies was shown by Grifoni et al. 2020 [5].

5-aminolevulinic acid (5-ALA) is a natural substance found in mitochondria that is significantly involved in producing Adenosine Tri-phosphate and, thus, in energy provision [6,7]. As a phosphate salt and in combination with sodium ferrous citrate (SFC), it has been on the Japanese market since 2010 as an over-the-counter dietary supplement for faster recovery after sports activities, among other things. In vitro tests have shown that the targeted and supplemental intake of 5-ALA-phosphate + SFC can strengthen mitochondrial functions in aged cells [8]. Hu et al. [9] showed in a melanoma model that 5-ALA-phosphate + SFC appears to be an important metabolic regulator in depleted T cell metabolism. Thus, it can be speculated that the administration of the dietary supplement 5-ALA-phosphate + SFC may positively influence the immune response by enhancing immune cells through improved energy availability. Recent preclinical evidence supports the anti-viral effect of 5-ALA-phosphate against the Classical Swine Fever virus and different variants of SARS-CoV-2 [10,11].

Because of the strong correlation with SARS-CoV-2-specific T cell responses, the measurement of IgG titer appears to be an appropriate parameter to investigate the supportive potential of the 5-ALA-phosphate + SFC supplement on the immune system, and in this study, specifically on vaccination support to achieve sustained immunity against viruses.

In this study, we hypothesized that the administration of 5-ALA-phosphate + SFC to subjects vaccinated against COVID-19 could enhance the immune system, leading to re-activation and an increase in the vaccination response shown in the IgG titer. However, as it was planned as a pilot study, the primary objective was to evaluate safety and tolerability, especially in terms of undesired immune reactions, when 5-ALA-phosphate + SFC was administered together and up to several weeks after vaccination.

Our study is considered a novel approach because, on the one hand, the immune-supporting effect of a food supplement after repeated vaccinations against COVID-19 was investigated; on the other hand, antibody concentrations were used as a quantitatively evaluable parameter. If the antibody levels actually remain higher for longer than without the supplement, this could possibly lead to greater protection against infections or delay the need for booster vaccinations or render them. The latter would also be beneficial for the healthcare systems. Moreover, the 5-ALA + SFC food supplement may be a cost-effective alternative to immune system-supporting drugs.

## 2. Materials and Methods

### 2.1. Study Design and Subject Characteristics

The present study was an open-label, two-arm interventional exploratory study to evaluate the safety and efficacy of 5-ALA-Phosphate + SFC during the vaccination of subjects against SARS-CoV-2. This study was approved and monitored by the Institutional/Independent Ethics Committee to safeguard the rights, safety, and well-being of all trial participants and was conducted following good clinical practice in compliance with the Declaration of Helsinki, revised by the World Medical Association 59th General Assembly, Seoul, 2008; the International Conference on Harmonization (ICH) recommendation on good clinical practice—E6(R2), 2016; and National Ethical Guidelines for Biomedical and Health Research involving Human Participants, 2017, issued by the Indian Council of Medical Research, India. All patients provided written informed consent. A clinical monitor regularly checked data integrity and quality during on-site monitoring visits.

The study was conducted from March to June 2022 in Maharashtra and Rajasthan, India, directly after the 3rd wave of corona infections in India. At least 250 subjects were screened, of whom 200 subjects were recruited. The recruited subjects were randomized in a ratio of 1:1 within 2 arms (5-ALA-phosphate + SFC and Control) to obtain not more than 160 completed subjects (80 in each arm), keeping 20% dropouts or lost to follow-up.

Only male and female subjects aged between 18 years and 70 years with documented proof of the 1st/2nd dose of vaccination were included in the study.

Almost half of the participants in both arms had a positive COVID-19 history. Covishield was the vaccine of choice for most participants in both arms (87 and 85 subjects received Covishield, and 12 and 16 subjects received Covaxin in the 5-ALA-phosphate + SFC and control arms, respectively). In both arms, almost equal numbers of participants were vaccinated with the second and third doses of vaccination during the study. The oxygen saturation level of the participants ranged from 93 to 100%. In both arms, most participants had serum IgG levels <5000 U/mL at baseline.

Subjects were excluded from trial participation when they suffered from anemia (defined as Hemoglobin level: male <12 g/dL, females <11 g/dL) or their liver enzymes Alanine Aminotransferase (ALT) and Aspartate Aminotransferase (AST) were clinically relevantly elevated (more than 2.5 times the upper limit). Moreover, subjects with an acute symptomatic COVID-19 infection indicated by fever, dry cough, and severe respiratory distress were excluded from the trial. During screening, the subjects were asked whether they had suffered from a COVID-19 infection in the past. However, a positive answer was not considered an exclusion criterion, but the information was only used for group comparison. The complete list of inclusion and exclusion criteria can be found at ClinicalTrials.gov (ID: NCT05234346).

The study participants were supplemented with 5-ALA phosphate + SFC for 21 days. Two capsules were administered in the morning after breakfast and 1 capsule in the evening after dinner orally with water, resulting in a daily dose of 150 mg 5-ALA-Phosphate + SFC (each capsule contains 50 mg 5-ALA-Phosphate + 28.68 mg SFC). The safety of the study product was determined by monitoring the occurrence of adverse events (AEs). In addition, the efficacy of the study product was evaluated by comparing the absolute change in geometric mean titer (GMT) of IgG levels against COVID-19 spike protein, the 5-Dimension Quality of Life Questionnaire from the EuroQuol group (EQ-5D-5L) score, the 5-dimension well-being questionnaire from WHO (WHO-5 well-being) score, and the Visual Analog Scale (VAS) for pain and fatigue.

The total study duration was 24 days, with an intervention period of 21 days. This treatment duration was chosen as in a former study conducted in COVID-19 patients, 5-ALA-Phosphate + SFC was administered in the same dose for 21 days as maintenance treatment. The treatment duration and dose were evaluated as safe. Moreover, in patients with severe COVID-19 symptoms, significantly lower levels of C-reactive protein, procalcitonin, and interleukin-6 compared to baseline were observed, which were evaluated as signs of efficacy [12]. Therefore, a treatment period of 21 days was considered in our pilot study as an appropriate scheme for evaluating the proof of concept.

**Figure 1:**
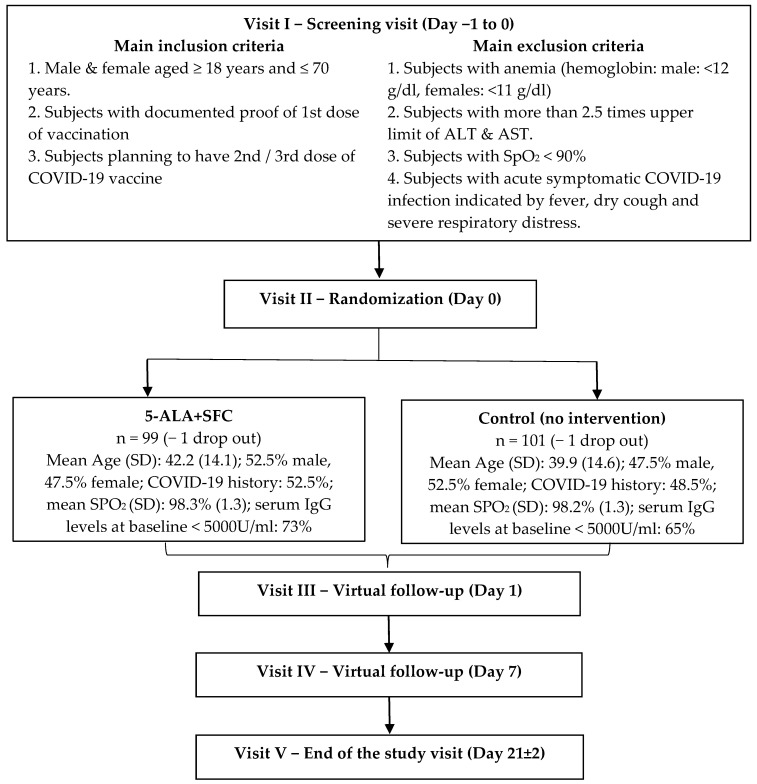
Study flow chart.

A detailed flowchart of time points at which safety, efficacy and other assessments should be conducted, as well as the key inclusion and exclusion criteria and group-specific characteristics, is shown in Figure 1.

Alanine Aminotransferase (ALT), Aspartate Aminotransferase (AST), peripheral capillary oxygen saturation (SpO_2_). In addition, the visit-specific study schedule is provided in Table 1.

### 2.2. Prior and Concomitant Therapy

All types of concomitant medication as prescribed by the physicians/investigators were allowed in this study except for experimental substances/investigational medicinal products that were not on the market and applied in the context of another clinical study. Moreover, taking vitamin D supplements during the trial was prohibited. Vitamin B and C supplements were allowed during the study.

Details of prior medications taken within 28 days before the start of screening and throughout the study and concomitant medications taken during the study, including start date, stop date or ongoing, the reason for use, dose, route, and frequency, were collected.

### 2.3. Measurement of IgG Levels

Elecsys Anti-SARS-CoV-2 S kit from Roche Diagnostics, 68305 Mannheim, Germany, was used to measure the levels of IgG post-vaccination at the baseline and at the end of the study (day 21). The double antigen-sandwich assay of the collected blood samples was performed as per the manufacturer’s guidelines and standard operating procedures.

The unit for the antibodies is a company unit with the following definition: Internal Roche standard for anti-SARS-CoV-2-S consisting of monoclonal antibodies. 1 nM of these antibodies corresponds to 20 U/mL of the Elecsys^®^ Anti-SARS-CoV-2 S assay.

### 2.4. Statistical Methods

Data processing, tabulation of descriptive statistics, and calculation of differences between groups were performed using the Statistical Package of SPSS, IBM, Version 27.0 (New York, NY, USA).

Continuous variables were subjected to normality testing using the Shapiro–Wilk test, and if found normal, then analyzed using parametric tests, and non-normal data were analyzed using non-parametric tests for hypothesis testing. Demographic and other baseline parameters were compared between the arms for differences using the Mann–Whitney U test (non-parametric, 2 independent-sample test) for continuous data and the chi-square test for categorical data.

Incidence of adverse product reaction and solicited vaccine-related local and systemic reactions were analyzed using the chi-square test/Fisher exact test for differences between the two arms. A listing of all AEs (original term) has been generated, including severity, seriousness, causality, action taken, and outcome. The number and percentage of participants with AEs were summarized using MedDRA terms grouped by preferred terms and system organ classes. Furthermore, the number of events was calculated. Other secondary outcomes were compared between the two arms using the Mann–Whitney U test and ANCOVA with intervention as a factor and baseline as a covariate where appropriate. The within-group comparison between the baseline and post-baseline assessments was the Wilcoxon signed-rank test or Friedman’s test, as appropriate. A *p*-value <0.05 and 95% confidence intervals were considered for statistical significance, and a two-tailed hypothesis was tested.

### 2.5. Primary Objective and Endpoints

The primary objective of this study was safety with the following primary endpoints:

- Number of participants with adverse product reactions as per Common Terminology Criteria for AE grading;
- Number of participants with solicited vaccine-related local and systemic adverse reactions (ARs).

### 2.6. Secondary Objective and Endpoints

The secondary objective was the efficacy of 5-ALA-phosphate + SFC in enhancing the immune system; the secondary endpoints were measured by the change in the following parameters as compared to the control arm:

- Absolute change in GMT of IgG levels against spike protein of SARS-CoV-2.
- EQ-5D-5L score.
- VAS for pain and fatigue.
- WHO-5 well-being questionnaire score.

## 3. Results

It was observed that both 5-ALA-phosphate + SFC and control arms showed a remarkable increase in the WHO-5 well-being index scores, indicating the improvement in the well-being of the participants over 21 days of the study period. The improvement was numerically greater in the intervention arm (mean change from baseline: +7.63) than in the control arm (mean change from baseline: +4.04); however, no statistically significant difference was observed between the two arms (*p* = 0.75).

Both 5-ALA + SFC and control arms showed a remarkable reduction in the VAS scores for pain at the injection site over the study period, with no pain at the end of the intervention. However, no significant difference was observed between the two arms except on Day 6, when a statistically significant difference (*p* = 0.04) was noted in the two arms, with the control arm having a better reduction in VAS scores compared to the 5-ALA-phosphate + SFC arm.

The generalized overall fatigue VAS scores showed a remarkable reduction as well in the 5-ALA-phosphate + SFC and control arms over the study period from baseline scores. However, no statistically significant difference was observed between the two arms at any single time point.

### 3.1. Primary Outcome—Safety: Adverse Product Reactions

One hundred ninety-eight participants reported AEs during the study, out of which 97 were in the 5-ALA-phosphate + SFC arm and 101 in the control arm. However, none of the AEs was assessed by the investigators as related to the study product.

The odds of reporting no AEs were 5.2× higher in the intervention group (95% CI: 0.25–110) (Table 2).

During the study duration, 517 out of 537 reported AEs were found to be related to the vaccine administration, as presented in Table 3. The two arms were comparable in the number of AEs reported after vaccine administration (with *p*-values from 0.38 to 0.75). Except for fatigue, a statistically significant difference between the arms after vaccination has been observed, with 86 cases in the 5-ALA + SFC arm and 95 cases in the control arm (*p* = 0.08).

Twenty AEs reported after vaccine administration were reported to be unrelated to either the vaccine or the study product (Table 4). All these 20 events occurred after a substantial amount of time of starting the IP. Most of the events were resolved with symptomatic treatment with sequelae. The participants could complete the treatment duration thereafter uneventfully. Hence, all the events were judged by the investigator as not related to the IP. Also, no serious adverse events were reported during the study.

### 3.2. Secondary Outcome—Efficacy: Change in GMT of IgG Levels Against COVID-19 Spike Protein

**Figure 2:**
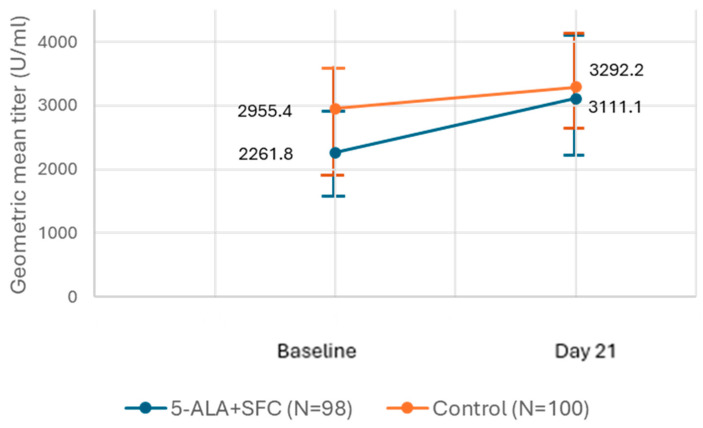
GMT (U/mL) for IgG antibodies.

The GMT is the preferred statistic for presenting the immune response to a vaccine [13]. GMT of IgG antibodies increased from the baseline to day 21 in both arms. However, the increase was 850 U/mL in the intervention arm, remarkably higher than in the control arm, in which the increase was 337 U/mL (Figure 2). However, due to the high and overlapping confidence intervals, the change was not statistically significantly different between the arms.

The baseline and day 21 GMT of IgG antibodies are presented in the above figure. The X-axis represents the time points considered for analysis (baseline and day 21) and the Y-axis represents GMT. The GMT of IgG in both study arms (5-ALA + SFC: blue; control: orange) increased from the baseline values.

**Figure 3:**
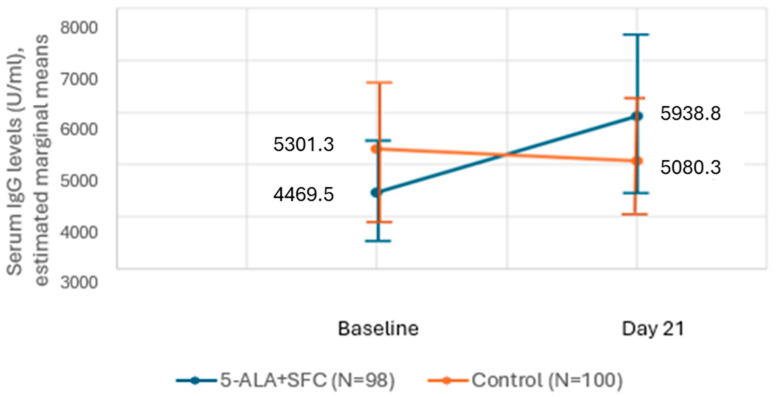
Serum IgG levels (U/mL) as estimated marginal means in two arms.

Figure 3 shows the serum IgG levels as estimated marginal means. Whereas the levels increased from baseline to day 21 by 1469 U/mL in the intervention arm, the levels decreased in the control arm by 221 U/mL. However, these values also have a high variation in the groups indicated by the overlapping confidence intervals, and therefore, the difference between the two groups was not statistically significant.

Also, the calculated geometric mean fold rise (GMFR) from baseline to day 21 was 1.38 higher in the intervention arm compared to the control arm, in which the calculated value was 1.11 (Table 5). Although the results were not statistically significant here either, a strong signal can be derived from the various evaluations that the study product increased the IgG antibody titer in the intervention arm.

The baseline mean serum IgG level was less than 5000 U/mL in the intervention arm and more than 5000 U/mL in the control arm; however, this difference between the arms was statistically insignificant (Table 6). On day 21, there was an increase in the mean serum IgG values in the intervention arm and a decrease in the control arm. The difference between the two arms was not significant. On the contrary, the median values of IgG levels show an increase in both arms on day 21 from their baseline values.

Mean and median change in both arms shows an increase in the intervention arm, while there was a decrease or no change in the control arm. However, the change from the baseline and between the groups was not clinically or statistically significant.

Domain-wise summary of EQ-5D-5L showed that no statistically significant difference was observed between the two arms at any time point in any of the domains of quality of life during the study.

#### Subgroup Analyses

Based on the values of serum IgG observed at baseline in the study population, three subgroups—<1000, 1000 to <5000, and ≥5000 U/mL—were formed, and analyses were performed with GMT and arithmetic means of serum IgG levels.

Further, age- and COVID-19 history-based categories were formed, and serum IgG levels were analyzed, as presented in Tables S1 and S2.

Based on the values of serum IgG observed at baseline in the study population, three subgroups—<1000, 1000 to <5000, and ≥5000 U/mL—were formed, and analyses were performed with GMT and arithmetic means of serum IgG levels.

Further, age- and COVID-19 history-based categories were formed, and serum IgG levels were analyzed.

In the subgroup with <1000 U/mL IgG levels, a statistically significant increase in the GMT of the IgG antibodies from baseline to day 21 in both the 5-ALA + SFC and control arms was observed.

In the subgroup with <5000 U/mL baseline IgG level, a statistically significant increase in the GMT of the IgG antibodies in both 5-ALA-phosphate + SFC and the control arm was observed. On the contrary, in the subgroup having ≥5000 U/mL baseline IgG levels, a reduction was observed in GMT of IgG antibodies in both 5-ALA-phosphate + SFC and the control arm; however, this reduction was statistically significant only in the control arm (Table S1).

An increase in the GMT of IgG antibodies was observed from baseline across the two age categories, and this increase was statistically insignificant in both arms. However, when the age categories were further divided based on the COVID-19 history of the participants, it was noted that the control arm having negative COVID-19 history (COVID-19 -ve)and <50 years of age experienced a significant reduction in GMT of IgG antibodies.

With subgroup analysis presented as arithmetic means, IgG levels show no statistically significant difference between the two groups after 21 days of intervention. However, some trends were observed. Subjects with a low baseline IgG antibody level (<1000 U/mL) in the intervention group showed, after 21 days of 5-ALA-phosphate + SFC administration, an approximately 2-fold higher IgG antibody titer level (4414 U/mL) than comparable subjects in the control group (2411 U/mL). There was an increase in the IgG levels from baseline to day 21 in the participants who had baseline IgG levels < 5000 U/mL, while those with baseline IgG levels ≥ 5000 U/mL had a reduction in mean (from 11,147 U/mL at baseline to 9400 U/mL after 21 days in the intervention group; from 10,902 U/mL at baseline to 6000 U/mL after 21 days in the control group) and median IgG (from 9650 U/mL at baseline to 7901 U/mL after 21 days in the intervention group; from 7130 U/mL at baseline to 4649 after 21 days in the control group) on day 21 from the baseline levels.

In the subgroup analysis based on age and COVID-19 history, it was observed that those who were <50 years of age, had a positive COVID-19 history (COVID-19 +ve), and received 5-ALA-Phosphate + SFC reported a very small decrease in the median IgG levels (from 2500 U/mL at baseline to 2393 U/mL on day 21), while those who received no intervention showed an increase in their median IgG levels (from 2500 U/mL at baseline to 4207 U/mL on day 21). On the contrary, those who had a negative COVID-19 history (COVID-19 -ve) and received 5-ALA-phosphate + SFC reported a very small increase in the median IgG levels (from 3182 U/mL at baseline to 3783 U/mL on day 21), and those who received no intervention demonstrated a decrease in median IgG level (from 3631 U/mL at baseline to 2750 U/mL on day 21).

Participants aged ≥50 years showed an increase in their median IgG levels irrespective of their COVID-19 history; however, the difference between the two groups was statistically insignificant.

## 4. Discussion

The SARS-CoV-2 virus enters human host cells by binding the spike protein to the receptors of angiotensin-converting enzyme 2 [14,15,16]. Thereafter, antigen-presenting cells like dendritic cells, macrophages and B cells will present antigens on their surface to activate T cells [17].

Vaccines such as Covishield and Covaxin are designed to induce human cells to produce spike proteins, and as a measure of the immune system, the body responds by producing neutralizing antibodies [18], which are suggested to be critical for immune protection [19]. However, study results show that neutralizing antibody titer decreases with time after a maximum was recognized after one month of the second vaccination with Covishield [20]. Emerging evidence has also suggested that SARS-CoV-2-specific CD4+ and CD8+ T cells, in coordination with neutralizing antibodies, are required to generate protective immunity against SARS-CoV-2 [21]. Adjuvants such as aluminum-based salts, Toll-like receptor agonists, emulsions, and other novel adjuvants have distinctive physicochemical properties, which can be significant in regulating the strength, duration, and types of immune responses [22,23,24]. Previous preclinical studies have shown that vaccine adjuvants such as alum produced a geometric mean titer (GMT) twice as high as the non-adjuvanted vaccine [25]. Based on the previous evidence [10,11], it was hypothesized that the administration of 5-ALA-phosphate + SFC in subjects vaccinated against COVID-19 could enhance the targeted function of the immune system, which might lead to re-activation and/or increase the vaccination response. The current study was designed to test the antibody response potentiation after the second as well as booster doses of vaccines (Covishield/Covaxin).

The study results demonstrated that the combination of 5-ALA-phosphate and SFC, when administered to recently vaccinated individuals, was completely safe, as no product-related AEs were reported during the study. It was also noted that adding 5-ALA-phosphate + SFC to the vaccines resulted in five times higher odds of having no AE post-vaccination. Most of the AEs reported post-vaccination were related to the vaccine, mild in severity, resolved without complications, and were comparable to AEs reported in previous studies on the vaccine [26,27].

The safety of the product has also been demonstrated in several clinical studies on hyperglycemic subjects and patients with type 2 diabetes mellitus. Exposure ranged from 1 day to 12 weeks, and the doses administered ranged from 5 mg to 1500 mg of 5-ALA-phosphate and 2.87 mg to 2351 mg pf SFC [28,29,30,31].

In addition to the proven good tolerability, our study showed at least a strong signal that the dietary supplement 5-ALA phosphate + SFC can enhance vaccination success, as shown by the IgG titer measurements. The GMT in IgG increase from baseline to day 21 was 850 U/mL in the intervention arm, remarkably higher than in the control arm, in which the increase was 337 U/mL. Moreover, the estimated marginal means of IgG increased from baseline to day 21 by 1469 U/mL in the intervention arm, whereas the levels decreased in the control arm by 221 U/mL. And the GMFR in the 5-ALA-phosphate + SFC arm was greater (1.38) than in the control arm (1.11) without intervention after 21 days. Although the results were not statistically significant, a clear trend can be seen that the dietary supplement 5-ALA-phosphate + SFC increased IgG antibody titer more than in the control group, in which the subjects received vaccination only. This trend can be especially noticed in the subgroup analysis based on baseline serum IgG levels, age group, and/or COVID-19 history (Table S2). Subjects with a low baseline IgG antibody level (<1000 U/mL) reached in the intervention group after 21 days of 5-ALA-phosphate + SFC administration an approximately 2-fold higher IgG antibody titer level (4414 U/mL) than comparable subjects in the control group (2411 U/mL). Therefore, supplementation might be particularly recommended in subjects with low baseline IgG titers. However, this is not limited to this subgroup, as concomitant administration is generally safe, and no contrary trend was observed in other baseline titer categories. For instance, a positive impact can be demonstrated in subjects with high IgG antibody levels (>/=5000 U/mL), in whom high IgG antibody levels persisted longer in the intervention group (from 11,147 U/mL at baseline to 9400 U/mL after 21 days of supplementation) than in the control group (from 10,902 U/mL at baseline to 6000 U/mL after 21 days without supplementation). The same trend can be noticed in subjects with an age of ≥50 years and a positive COVID-19 history. Again, the administration of 5-ALA-phosphate + SFC kept the subjects at a high IgG antibody level for longer. Thus, a slight increase was observed in the intervention group (+186 U/mL), while there was even a remarkable decrease in IgG antibodies (−1124 U/mL) in the group with vaccination alone.

Although the results are not statistically significant, it can be stated that 5-ALA-phosphate + SFC is a potentially beneficial supplement when taken after COVID-19 vaccination. The evaluation of subject recordings from established quality of life questionnaires (EQ-5D-5L and WHO well-being) leads to the statement that 5-ALA-phosphate + SFC administration did not limit well-being, which is already a quite important result.

The VAS scores for pain at the injection site and the generalized overall fatigue also showed no statistically significant difference between the two arms. However, this can be envisioned as a positive outcome considering that the immune-boosting potential of 5-ALA-phosphate + SFC is not accompanied by increased vaccination-related adverse events like pain at the injection site.

The evaluation of the subject questionnaires showed that the administration did not limit the well-being of the participants, which confirms the excellent tolerability of this food supplement. However, further studies would be recommended for verification of the results.

There are some limitations to the interpretation of the study results. Owing to the high individual variation in the IgG titer response after vaccination, the number of patients was too low to obtain significant differences between the intervention and the control group. Moreover, this variation was most probably so high due to the widespread inclusion criteria for known IgG titer-dependent requirements for the patient population:

− A second or third dose of vaccine was allowed and may vary the IgG titer response.
− The vaccine was not limited to one type: the viral vector, Covishield, and the inactivated vaccine, Covaxin, were allowed, which might lead to different responses in vaccine-related AEs and IgG titer.
− Subjects could have suffered from a COVID-19 infection in the past, which would also impact the IgG titer response.
− Subjects aged ≥18 years and ≤70 years were eligible to participate in this study. As age could be decisive for the IgG titer response, an even distribution of subjects to predefined age groups would have been ideal.

Even stratification with regard to these influencing factors did not lead to any significant differences between the groups, as the number of patients in the subgroups was even lower, and the dispersion of values remained high. Nonetheless, the primary objective was patient safety, which was successfully demonstrated, and signs of efficacy were shown, which, in the authors’ opinion, were sufficient for this pilot study. If it is decided that a development program for 5-ALA as an immune booster for vaccination should be initiated, then further studies should be conducted with a well-defined patient population and a sufficient number of subjects according to a statistical sample size calculation.

## 5. Conclusions

5-ALA-phosphate + SFC (150 mg daily) can be safely given together with (vaccines against SARS-CoV-2 (Covishield/Covaxin, viral vector vaccines) and up to 21 days thereafter. Therefore, the primary objective of this study was met. Moreover, the results for the secondary endpoint ‘Change in Immunoglobulin G levels’, although statistically not significant, provided strong signals of immune-boosting properties of 5-ALA-phosphate + SFC. Therefore, IgG antibody titer increases were remarkably greater in the intervention group than in the control group. The evaluation of the subject questionnaires showed that the administration did not limit the well-being of the participants, which confirms the excellent tolerability of this food supplement. However, further studies would be recommended for verification of the results and to investigate the transferability to other types of vaccines and target indications, e.g., influenza.

## Figures and Tables

**Table 1:** Study visit schedule.

Study Day	Screening/Randomization Visit (Day 0)	Virtual Visit (Day 1)	Virtual Visit (Day 7)	End of Study Visit (Day 21 ± 2)
Visits	**1**	**3**	**4**	**5**
Informed consent	X			
Medical history	X			
Medication history	X			
Study-specific history	X			X
Demographics data (age, gender)	X			
Anthropometrics data (height and weight)	X			
Vitals (PR, BP, SpO2, and body temperature)	X			X
Urine pregnancy test	X			X
Clinical examination	X			X
Concomitant medication				X
RT-PCR		X		X
Blood sample collection (Hemoglobin, ALT and AST)	X			
Inclusion/exclusion criteria	X			
Randomization process	X			
2**nd** dose/booster dose	X			
Blood sample collection (IgG)	X			X
EQ-5D-5L	X	X	X	X
VAS (pain and fatigue)	X	X		X
WHO-5 well-being questionnaire	X	X	X	X
IP dispensing	X			
IP diary dispensing	X			
Daily diary dispensing	X			X
IP reconciliation				X
AE/SAE	X			
ADR	X			

NOTE 1: Subjects reporting symptoms of COVID-19 underwent Polymerase Chain Reaction (PCR) testing at any time during the study period. Those reporting symptoms of COVID-19 and a positive PCR test during the study period were only evaluated for safety parameters. The result of the PCR test did not hamper study product consumption, i.e., PCR-positive subjects continued the study product consumption for 21 days. NOTE 2: The pain Visual Analog Scale (VAS) was assessed for the site of injection, and the fatigue VAS was assessed for generalized fatigue post-vaccination daily for 7 days. Pulse Rate (PR), Blood Pressure (BP), Reverse Transcription-PCR (RT-PCR), Immunoglobulin G (IgG), 5-Dimension Quality of Life Questionnaire from EuroQuol group (EQ-5D-5L), 5-dimension well-being questionnaire from World Health Organization (WHO-5 well-being questionnaire), study/investigational product (IP), adverse event (AE), serious adverse event (SAE), adverse drug reaction (ADR).

**Table 2:** Number of patients reporting adverse events (Full Analysis Set (FAS)).

Category	5-ALA + SFC + Vaccine (*n* = 99)		Vaccine (*n* = 101)		*p*-Value
	*n*	%	*n*	%	
No AE	2	1.0	0	0.0	0.152
AE	97	98.0	101	100.0	
	Odds Ratio (Arm 1/Arm 2), 5.205 (95% CI 0.246–109.81)				

Note 1: AEs have been reported as [*n*] and [%], where *n* = unique subject count with AEs (subjects experiencing multiple AEs within the same preferred term are counted only once within that preferred term). % as a percentage of subjects with AEs and n as the Total Event Count. Note 2: All percentages are based on the number of participants in the respective study arm in the FAS population. Note 3: The *p*-value has been calculated using the odds ratio test.

**Table 3:** Adverse events related to vaccine administration in two arms (FAS).

	Preferred Term	5-ALA + SFC (*n* = 99)		Control (*n* = 101)		Total *n* = 200		*p*-Value
		*n* (%)	*n*	*n*(%)	*n*	*n* (%)	*n*	
Local	Injection site erythema	0 (0.0%)	0	4 (4.0%)	4	4 (2.0%)	4	NE
	Injection site pain	96 (97.0%)	100	99 (98.0%)	101	195 (97.5%)	201	0.6812 (F)
	Injection site rash	0 (0.0%)	0	1 (1.0%)	1	1 (0.5%)	1	NE
Systemic	Asthenia	5 (5.1%)	5	2 (2.0%)	2	7 (3.5%)	7	0.2767 (F)
	Diarrhea	1 (1.0%)	1	0 (0.0%)	0	1 (0.5%)	1	NE
	Fatigue	86 (86.9%)	106	95 (94.1%)	108	181 (90.5%)	214	0.0829 (C)
	Headache	8 (8.1%)	8	7 (6.9%)	7	15 (7.5%)	15	0.7575 (C)
	Myalgia	7 (7.1%)	7	5 (5.0%)	5	12 (6.0%)	12	0.5279 (C)
	Nausea	2 (2.0%)	2	1 (1.0%)	1	3 (1.5%)	3	0.6193 (F)
	Pyrexia	31 (31.3%)	31	26 (25.7%)	26	57 (28.5%)	57	0.3829 (C)
	Somnolence	1 (1.0%)	1	0 (0.0%)	0	1 (0.5%)	1	NE
	Vomiting	1 (1.0%)	1	0 (0.0%)	0	1 (0.5%)	1	NE
	Total	97 (98.0%)	262	100 (99.0%)	255	197 (98.5%)	517	0.6193 (F)

Note 1: AEs have been reported as [*n* (%) *n*], where *n* = unique subject count with AEs (subjects experiencing multiple AEs within the same preferred term are counted only once within that preferred term). % as a percentage of subjects with AEs and n as the Total Event Count. Note 2: All percentages are based on the number of participants in the respective study arm in the FAS population. Note 3: The *p*-value has been calculated using the chi-square (C)/Fisher’s exact (F) test.

**Table 4:** Adverse events unrelated to study products in either arm (FAS dataset).

	Preferred Term	5-ALA + SFC (*n* = 99)		Control (*n* = 101)		Total (*n* = 200)		*p*-Value
		*n* (%)	*n*	*n*(%)	*n*	*n* (%)	*n*	
Systemic	Abdominal pain	0 (0.0%)	0	1 (1.0%)	1	1 (0.5%)	1	NE
	Cough	1 (1.0%)	1	0 (0.0%)	0	1 (0.5%)	1	NE
	Dehydration	0 (0.0%)	0	1 (1.0%)	1	1 (0.5%)	1	NE
	Diarrhea	1 (1.0%)	1	1 (1.0%)	1	2 (1.0%)	2	>0.9999 (F)
	Fatigue	2 (2.0%)	2	1 (1.0%)	1	3 (1.5%)	3	0.6193 (F)
	Headache	0 (0.0%)	0	3 (3.0%)	3	3 (1.5%)	3	NE
	Myalgia	0 (0.0%)	0	4 (4.0%)	4	4 (2.0%)	4	NE
	Rhinorrhoea	1 (1.0%)	1	1 (1.0%)	1	2 (1.0%)	2	>0.9999 (F)
	Somnolence	0 (0.0%)	0	1 (1.0%)	1	1 (0.5%)	1	NE
	Vomiting	1 (1.0%)	1	1 (1.0%)	1	2 (1.0%)	2	>0.9999 (F)
	Total	5 (5.1%)	6	12 (11.9%)	14	17 (8.5%)	20	0.0833 (C)

Note 1: Adverse events have been reported as [*n* (%) *n*], where *n* = unique subject count with adverse events (subjects experiencing multiple adverse events within the same preferred term are counted only once within that preferred term). % as a percentage of subjects with AEs and *n* as the Total Event Count. Note 2: All percentages are based on the number of participants in the respective study arm in the FAS population. Note 3: The *p*-value has been calculated using the chi-square (C)/Fisher’s exact (F) test; NE = not evaluable.

**Table 5:** GMFR for serum IgG levels.

	GMFR	95% CI	*p*-Value
5-ALA + SFC (N = 98)	1.38	(0.92–2.06)	0.135
Control (N = 100)	1.11	(0.80–1.55)	0.953

Notes: *p*-value was calculated using the Wilcoxon test (within-group comparisons). Abbreviations: CI = confidence interval, GMFR = geometric mean fold rise, N = number of participants.

**Table 6:** Serum IgG levels (U/mL) in two groups at baseline and day 21 (ITT dataset).

	5-ALA + SFC (N = 98)				Control (N = 100)				
	Mean (SD)	Median	95% CI	Min.–Max.	Mean (SD)	Median	95% CI	Min.–Max.	*p*-Value
Baseline	4469.5 (4853.0)	2500.0	3496.6–5442.5	7.44–23074	5301.3 (6614.1)	3248.0	3988.9–6613.7	8.3–42,303	0.197
Day 21	5938.8 (7528.2)	3427.5	4429.5–7448.1	9.5–47525	5080.3 (5437.3)	3898.0	4001.4–6159.2	10.18–41,266	0.692
Change	1469.3 (7381.1)	54.5	−10.6–2949.1	−22,271.50–36,635.60	−221.0 (6336.1)	0.0	−1478.2–1036.3	−38,919.0–18,112.0	0.291

Notes: The *p*-value is calculated using the Mann–Whitney U test for between-group comparison. Abbreviations: CI = confidence interval, N = number of participants, SD = standard deviation.

## Data Availability

Most data are contained within the article. Additional data needed could be retrieved from the study report.

## Supplementary Materials

The following supporting information can be downloaded at https://www.mdpi.com/article/10.3390/life15060953/s1. S1: Subgroup analysis of GMT of serum IgG levels; S2: Summary and change for serum IgG in different sub-groups.

## Abbreviations

The following abbreviations are used in this manuscript:

5-ALA	5-aminolevulinic acid
SFC	sodium ferrous citrate
AE	adverse event
IgG	Immunoglobulin G
WHO	World Health Organization
PHEIC	Public Health Emergency of International Concern
mRNA	messenger ribonucleic acid
IEC	Institutional/Independent Ethics Committee
WMA	World Medical Association
ICH	International Conference on Harmonization
GCP	good clinical practice
ICMR	Indian Council of Medical Research
ALT	Alanine Aminotransferase
AST	Aspartate Aminotransferase
GMT	geometric mean titer
VAS	Visual Analog Scale
PR	Pulse Rate
BP	Blood Pressure
SPO2	peripheral capillary oxygen saturation
RT-PCR	Reverse Transcription-Polymerase Chain Reaction
EQ-5D-5L	5-Dimension Quality of Life Questionnaire from EuroQuol group
IP	investigational product
SAE	serious adverse event
APR	adverse product reaction
FAS	Full Analyses Set
ITT	intention-to-treat
CI	confidence interval
GMFR	geometric mean fold rise
N	number of participants
SD	standard deviation
U	unit
