# Supplementary material for: 5-Aminolevulinic Acid Phosphate as an Immune System Enhancer Along with Vaccination Against SARS-CoV-2 Virus Infection: An Open-Label, Randomized Pilot Study
Source: Life (Basel). 2025 Jun 13;15(6):953. doi: 10.3390/life15060953 (PMC12194446; doi:10.3390/life15060953)
Supplement: Supplementary file 1 [file life-15-00953-s001.zip › Table S1.pdf]

Table S1. Subgroup analysis of GMT of serum IgG levels

Subgroups	N	Baseline	Day 21	p-value
A) IgG level				
<1000 U/ml				
5-ALA+SFC (N=98)	16	181.06	909.21	**0.010**
Control (N=100)	09	176.22	703.71	**0.038**
1000 to <5000 U/ml				
5-ALA+SFC (N=98)	56	2305.75	3219.95	**0.011**
Control (N=100)	55	2243.67	3348.07	**<0.001**
<5000 U/ml				
5-ALA+SFC (N=98)	72	1309.96	2431.13	**<0.001**
Control (N=100)	64	1568.86	2688.66	**<0.001**
≥5000 U/ml				
5-ALA+SFC (N=98)	26	10264.36	6158.96	0.073
Control (N=100)	36	9111.26	4719.09	**<0.001**
B) Age and COVID history				
Age <50 yrs.				
5-ALA+SFC (N=98)	57	2504.83	3223.01	0.517
Control (N=100)	66	3195.43	3239.67	0.279
Age <50 yrs. (COVID +ve)				
5-ALA+SFC (N=98)	24	2212.89	2607.73	0.886
Control (N=100)	29	3487.09	4070.10	0.554
Age <50 yrs. (COVID -ve)				
5-ALA+SFC (N=98)	33	2741.07	3759.85	0.586
Control (N=100)	37	2983.99	2709.10	**0.028**
Age ≥50 yrs.				
5-ALA+SFC (N=98)	41	1962.69	2961.92	0.115
Control (N=100)	34	2539.81	3396.74	0.114
Age ≥50 yrs. (COVID +ve)				
5-ALA+SFC (N=98)	28	1495.07	2076.40	0.537
Control (N=100)	20	2587.61	2684.03	0.312
Age ≥50 yrs. (COVID -ve)				
5-ALA+SFC (N=98)	13	3527.08	6365.48	0.055
Control (N=100)	14	2473.04	4755.23	0.241
All patients				
5-ALA+SFC (N=98)	98	2261.84	3111.09	0.135
Control (N=100)	100	2955.44	3292.24	0.953

Notes: p-value was calculated using the Wilcoxon test (within-group comparisons)

Abbreviations: GMT=geometric mean titer; N=Number of participants; -ve=negative; +ve=positive
