# Supplementary material for: 5-Aminolevulinic Acid Phosphate as an Immune System Enhancer Along with Vaccination Against SARS-CoV-2 Virus Infection: An Open-Label, Randomized Pilot Study
Source: Life (Basel). 2025 Jun 13;15(6):953. doi: 10.3390/life15060953 (PMC12194446; doi:10.3390/life15060953)
Supplement: Supplementary file 1 [file life-15-00953-s001.zip › Table S2.pdf]

Table S2. Summary and change for serum IgG in different sub-groups

Subgroups	5-ALA+SFC (N=98)				Control (N=100)				
	N	Mean (SD)	Median	95% CI	N	Mean (SD)	Median	95% CI	p-value
**A)** IgG level									
<1000 U/ml									
Baseline	16	396 (317.6)	378.5	226.7 - 565.2	9	419.3 (353.3)	523.7	147.7 - 690.9	0.865
Day 21	16	4810.7 (9458.1)	877.1	-229.2 - 9850.6	9	2830.3 (5380.4)	735.1	-1305.4 - 6966	0.821
Change from baseline	16	4414.7 (9455.2)	596.3	-623.6 - 9453.1	9	2411 (5284.4)	256	-1650.9 - 6473	0.651
1000 - <5000 U/ml									
Baseline	56	2532.9 (1070.5)	2350	2246.3 - 2819.6	55	2434.2 (942.3)	2500	2179.5 - 2688.9	0.883
Day 21	56	4639.9 (5407.7)	3006.5	3191.7 - 6088.1	55	4469.3 (3901.2)	3852	3414.7 - 5524	0.391
Change from baseline	56	2106.9 (5489.1)	347.5	636.9 - 3576.9	55	2035.1 (3878.4)	758	986.6 - 3083.6	0.286
<5000 U/ml									
Baseline	72	2058.1 (1307.5)	2017	1750.8 - 2365.3	64	2150.9 (1129.3)	2289.5	1868.8 - 2432.9	0.469
Day 21	72	4677.8 (6446.5)	2435.5	3163 - 6192.7	64	4238.8 (4129.3)	3649	3207.4 - 5270.3	0.414
Change from baseline	72	2619.8 (6569.7)	370.4	1076 - 4163.6	64	2088 (4056.7)	599	1074.6 - 3101.3	0.513
≥ 5000 U/ml									
Baseline	26	11147.5 (4837.2)	9650	9193.7 - 13101.3	36	10902 (8427.9)	7130	8050.4 - 13753.6	0.118
Day 21	26	9430.7 (9198.4)	7901	5715.4 - 13146	36	6576.3 (7024)	4649	4199.7 - 8952.8	0.087
Change from baseline	26	-1716.8 (8627.7)	-1154.5	-5201.6 - 1768	36	-4325.8 (7540.3)	-2817.5	-6877 - - 1774.5	0.325
B) Age and COVID history									

Age <50 yrs.									
Baseline	57	4410.0 (4794.3)	2500.0	3137.9 - 5682.1	66	5058.0 (4524.2)	3411.0	3945.9 - 6170.2	0.171
Day 21	57	6140.8 (8648.2)	3394.0	3846.1 - 8435.4	66	4487.4 (3876.3)	3824.5	3534.5 - 5440.4	0.970
Change from baseline	57	1730.7 (8003.2)	-4.0	-392.8 - 3854.3	66	-570.6 (4537.5)	-207.6	-1686.1 - 544.8	0.153
Age <50 yrs. (COVID +ve)									
Baseline	24	3988.7 (4328.0)	2500.0	2161.1 - 5816.2	29	4881.8 (4188.6)	2500.0	3288.5 - 6475.0	0.307
Day 21	24	6001.6 (9810.2)	2393.5	1859.2 - 10144.1	29	5348.4 (4437.8)	4207.0	3660.4 - 7036.5	0.198
Change from baseline	24	2013.0 (6326.7)	-68.5	-658.6 - 4684.5	29	466.6 (4900.1)	559.0	-1397.3 - 2330.5	0.929
Age <50 yrs. (COVID -ve)									
Baseline	33	4716.5 (5150.9)	3182.0	2890.0 - 6542.9	37	5196.2 (4823.6)	3631.0	3587.9 - 6804.4	0.309
Day 21	33	6242.0 (7854.1)	3783.0	3457.0 - 9026.9	37	3812.6 (3278.3)	2750.0	2719.6 - 4905.6	0.210
Change from baseline	33	1525.5 (9122.3)	50.0	-1709.1 - 4760.1	37	-1383.6 (4117.3)	-753.0	-2756.4 - - 10.8	0.094
Age ≥50 yrs.									
Baseline	41	4552.3 (4992.1)	2454.0	2976.6 - 6128.0	34	5773.4 (9517.0)	2500.0	2452.8 - 9094.1	0.758
Day 21	41	5658.0 (5708.5)	3573.0	3856.2 - 7459.8	34	6231.2 (7551.7)	4292.5	3596.3 - 8866.1	0.456
Change from baseline	41	1105.7 (6497.1)	357.4	-945.0 - 3156.4	34	457.7 (8897.6)	472.5	-2646.8 - 3562.3	0.750
Age ≥50 yrs. (COVID +ve)									

Baseline	28	4303.7 (5313.4)	2128.5	2243.4 - 6364.0	20	7200.3 (11822.0)	3281.5	1667.5 - 12733.2	0.286
Day 21	28	4490.1 (5528.0)	2421.0	2346.6 - 6633.7	20	6075.5 (8894.3)	4604.0	1912.8 - 10238.2	0.296
Change from baseline	28	186.4 (6806.0)	30.6	-2452.6 - 2825.5	20	-1124.8 (9326.6)	208.0	-5489.8 - 3240.2	0.738
Age ≥50 yrs. (COVID -ve)									
Baseline	13	5087.7 (4369.4)	3310.0	2447.3 - 7728.1	14	3735.0 (4247.9)	2382.0	1282.3 - 6187.7	0.264
Day 21	13	8173.4 (5461.7)	4638.0	4872.9 - 11473.9	14	6453.6 (5390.1)	3945.0	3341.4 - 9565.7	0.467
Change from baseline	13	3085.7 (5501.3)	2138.0	-238.8 - 6410.1	14	2718.6 (8029.5)	1761.5	-1917.5 - 7354.6	0.961

Notes: p-value was calculated using the Mann-Whitney U test. Abbreviations: CI=confidence interval, N= number of participants, SD=standard deviation; -ve=negative; +ve=positive
